# Relationship between the Mediterranean diet and risk of hepatic fibrosis in patients with non-alcoholic fatty liver disease: A cross-sectional analysis of the RaNCD cohort

**DOI:** 10.3389/fnut.2023.1062008

**Published:** 2023-02-22

**Authors:** Mahsa Miryan, Mitra Darbandi, Mozhgan Moradi, Farid Najafi, Davood Soleimani, Yahya Pasdar

**Affiliations:** ^1^Student Research Committee, Kermanshah University of Medical Sciences, Kermanshah, Iran; ^2^Nutritional Sciences Department, School of Nutrition Sciences and Food Technology, Kermanshah University of Medical Sciences, Kermanshah, Iran; ^3^Research Center for Environmental Determinants of Health (RCEDH), Health Institute, Kermanshah University of Medical Sciences, Kermanshah, Iran; ^4^Internal Medicine Department, School of Medicine Kermanshah University of Medical Sciences, Kermanshah, Iran; ^5^Cardiovascular Research Center, Kermanshah University of Medical Sciences, Kermanshah, Iran; ^6^Research Center of Oils and Fats, Kermanshah University of Medical Sciences, Kermanshah, Iran

**Keywords:** non-alcoholic fatty liver disease, fatty liver index, liver fibrosis, Mediterranean diet, PERSIAN cohort

## Abstract

**Background:**

Despite evidence supporting the beneficial effects of the Mediterranean diet (MedDiet) on hepatic steatosis in subjects with non-alcoholic fatty liver disease (NAFLD), the relationship of the MedDiet with hepatic fibrosis is as yet unclear. The aim of the present study was to explore this association in Iranian adults with NAFLD.

**Methods:**

This cross-sectional study included 3,325 subjects with NAFLD from the Ravansar Noncommunicable Disease (RaNCD) cohort. Dietary intake data were collected by a validated food frequency questionnaire (FFQ). The MedDiet score was computed based on a nine-point scale constructed by Trichopoulou et al. Fatty liver index (FLI) and fibrosis-4 (FIB-4) index were used to predict hepatic steatosis and fibrosis in the population. Multivariate regression models were applied to determine associations.

**Results:**

Subjects in the highest tertile of MedDiet score had a higher platelet and a lower weight, total cholesterol (TC), LDL-c, and FLI than those in the lowest tertile (*p*-value < 0.05). Adherence to the MedDiet was associated with a 7.48 (95%CI: 5.376 to 9.603; *p*-value: 0.001) × 10^3^/μl; −0.417 (95%CI: −0.819 to −0.014; *p*-value: 0.042) kg, −2.505 (95%CI: −3.835 to −1.175; *p*-value: 0.001) mg/dl; and −1.93 (95%CI: −2.803 to −1.061; *p*-value: 0.001) mg/dl change in platelet, weight, TC, and LDL-c for each SD increase in the score, respectively. A significant linear trend was observed in odds of hepatic fibrosis across the tertiles of the MedDiet score (P-trend: 0.008). This linear trend was attenuated but remained significant after the adjustment of the relevant confounders (P-trend: 0.032). Adherence to the MedDiet was independently associated with about 16% lower odds of having hepatic fibrosis in patients with NAFLD for each SD increase in the score.

**Conclusion:**

Adherence to the MedDiet characterized by a high intake of whole grains, fruits, vegetables, legumes, nuts, and fish was associated with a lower risk of having hepatic fibrosis in patients with NAFLD. Further studies are required to elucidate the causal relationship of observed association in individuals of all ages, ethnicities, and etiologies of hepatic steatosis.

## 1. Introduction

Nonalcoholic fatty liver disease (NAFLD) is characterized by the pathological accumulation of fat in the hepatocytes in the absence of excessive alcohol use and known secondary causes of steatosis such as hepatitis B and C. NAFLD is the most common chronic liver disease, affecting approximately 25 percent of the general population in the world (1). This disease represents a wide spectrum of histologic abnormalities in the liver from non–alcoholic fatty liver (NAFL) to non-alcoholic steatohepatitis (NASH). NASH is the more progressive form of NAFLD to fibrosis, cirrhosis, and hepatocellular carcinoma (2). The progression rate of fibrosis is 0.07 and 0.14 stages per year in NAFL and NASH patients, respectively (3). NAFLD has been predicted to become the leading cause of liver transplantation in the United States by 2030 (4). This disease also is independently associated with an increased risk of cardiovascular diseases (CVDs) (5). So far, no specific pharmaceutical agent has been approved by FDA for the treatment of NAFLD, and lifestyle modification remains the first-line treatment.

NAFLD is a consequence of the interaction between genes and various environmental factors. Diet as a modifiable environmental factor plays an essential role in NAFLD pathogenesis and progression. In this context, considerable research interest has been devoted to associations between dietary patterns and histological and clinical features of NAFLD. The MedDiet is a healthy dietary pattern associated with favorable health outcomes, mainly in relation to CVD. A high intake of whole grains, fruits, vegetables, seafood, beans, nuts, and olive oils characterizes this dietary pattern. Many observational studies revealed that adherence to the MedDiet is negatively associated with the serum levels of liver enzymes, the onset and severity of hepatic steatosis, and the presence of NASH in patients with NAFLD (6–8). In line with these efforts, several clinical trials showed that the MedDiet can substantially improve liver enzymes, hepatic steatosis, and insulin sensitivity in patients with NAFLD (9–11).

Despite evidence supporting the beneficial effect of the MedDiet on hepatic steatosis in patients with NAFLD, the relationship between the MedDiet and hepatic fibrosis is as yet unclear. To our knowledge, there is scarce literature on the association of MedDiet with hepatic fibrosis in NAFLD patients. The ATTICA cohort, which was conducted in Greece, showed a negative relationship between the MedDiet score and fibrosis-4 score (FIB-4), a proxy measure of hepatic fibrosis (12). Another study conducted on diabetic patients with NAFLD in Spain showed a lower adherence to MedDiet in patients with hepatic fibrosis (13). However, these results may not be applicable to Iranian adults due to different lifestyles, genetic predisposition to the disease, and confounding factors such as physical activities and alcohol consumption. Thus, we designed a population-based study to explore the association between adherence to MedDiet and the risk of hepatic fibrosis in Iranian adults with NAFLD based on valid non-invasive methods.

## 2. Subjects and methods

### 2.1. Study population and design

The present cross-sectional analysis was performed within the framework of the Ravanser Non-Communicable Disease (RaNCD) cohort. The RaNCD study is part of Prospective Epidemiological Research Studies in IRAN (PERSIAN). The RaNCD cohort was originally designed to determine the incidence of non-communicable diseases (NCDs), the main risk factors for NCDs, and the relationship between these risk factors and NCDs in the Kurdish ethnic population aged 35 to 65 years who permanently live in the Ravanser district of Kermanshah province, a region in western Iran. The RaNCD cohort recruited 10,065 participants between March 2015 and February 2017 who are still being followed up (14). The RaNCD cohort was conducted in accordance with the Declaration of Helsinki, and written informed consent was obtained from all participants. Of the original sample, we excluded participants who reported energy intake outside the normal ranges (600–3,500 kcal.day^−1^ for women and 800–4,200 kcal.day^−1^ for men), had missing relevant data, had the fatty liver index (FLI) < 60, had a history of alcohol intake, or had the secondary causes of steatosis (eg, viral hepatitis B and C). We also excluded subjects with missing data. Our final sample comprised 3,325 subjects with NAFLD defined through FLI. The present work was approved by the steering committee of the RaNCD and the ethics committee of Kermanshah University of Medical Sciences (IR.KUMS.REC.1401.223). The manuscript was approved by the members of the steering committee of the RaNCD, who assume responsibility for the integrity of the data and the overall content of the manuscript.

### 2.2. Non-invasive index of hepatic steatosis and fibrosis

The fatty liver index (FLI) is a well-known non-invasive and accurate predictor of hepatic steatosis in the general population. FLI is an algorithm of both anthropometric data and biochemical tests, including body mass index (BMI), waist circumference (WC), gamma-glutamyl transferase (GGT), and triglyceride (TG). We calculated FLI by the following formula (15):

$\begin{array}{l} \text{FL}\;\text{index}=\left(\begin{array}{l} e^{0.953\times \log}{{\;}_{e}}^{\left(\text{TG}\right)+0.139\times \text{BMI}+0.718\times \log}\\ {{\;}_{e}}^{\left(\text{GGT}\right)+0.053\times \text{WC}-15.745} \end{array}\right)\\ \;/\left(\begin{array}{l} 1+e^{0.953\times \log}{{\;}_{e}}^{\left(\text{TG}\right)+0.139\times \text{BMI}+0.718\times \log}\\ {{\;}_{e}}^{\left(\text{GGT}\right)+0.053\times \text{waist circumference}-15.745} \end{array}\right)\times 100 \end{array}$

FLI values are between 0 and 100, where an FLI ≥ 60 detects hepatic steatosis with an accuracy of 0.84 (95% confidence interval (CI) 0.81–0.87) (15). This index is widely used to detect hepatic steatosis in extensive epidemiological studies. FLI is also strongly associated with the severity of hepatic steatosis in the general population (16).

The fibrosis-4 (FIB-4) index is a non-invasive scoring system for predicting hepatic fibrosis in many large-scale epidemiological studies (12, 17). FIB-4 is an algorithm of biochemical tests and calculated using the following formula:


$$\text{FIB}-4=\text{Age}\;\left(\text{years}\right)\times \text{AST}\;\left(U/L\right)/\text{platelet}\;\left(10^{9}/L\right)\times {\text{ALT}}^{1/2}\;\left(U/L\right)$$

The cut-off points of FIB for predicting hepatic fibrosis were set at 1.05 in individuals aged ≤ 49 years, 1.24 in 50–59 years, 1.88 in 60–69 years, and 1.95 in ≥ 70 years with the area under the receiver operating characteristic curve (AUROC) of 0.917, 0.849, and 0.855, respectively (18).

### 2.3. Dietary data assessment and MedDiet score computation

Usual food intake was determined using the national Iranian food frequency questionnaire (FFQ) at the time of recruitment. This FFQ included questions about the frequency intake of 118 food items and appropriate standard portion sizes (e.g., a glass, cup, slice, teaspoon, tablespoon, spatula, cube, etc.) for each food item. Participants reported the average frequencies and portion sizes of consumed foods over the past year. In this study to decline the recall bias, the FFQ was taken from the participants by trained nutrition experts, and the participants were given enough time to remember the consumption of each food item. The FFQs were analyzed to obtain energy and nutrient intakes using the Nutritionist IV software (First Databank Inc., Hearst Corp., San Bruno, CA, United States) based on the U.S. Department of Agriculture food composition data. In nutritional epidemiological studies, subjects who under-reported (-3SD) or over-reported (+3SD) their energy intake based on FFQ analysis should be excluded from the study. According to a previous study, the under-reporting of energy intake in men and women is estimated at 800 and 600 kcal per day, and the over-reporting is estimated at 4200 and 3,500 kcal per day, respectively (19).

The MedDiet score was computed based on a nine-point scale constructed by Trichopoulou et al. This scale consists of nine dietary components: whole grains, fruits, vegetables, legumes, nuts, fish/seafood, monounsaturated to saturated fat ratio (MUFA/SFA) as healthy items; red and processed meats as unhealthy items; and alcohol as an item for which a moderate consumption was recommended. The food items included in each food group are shown in Table 1. Each component is assigned a value of 0 or 1 according to the sex-specific median of the studied population as the cut-off point. We measured each component (except for alcohol) in grams per 1,000 kilocalories to make intake independent of total energy intake (20). For healthy components, individuals with an intake at or above the median receive 1 point, otherwise they receive 0 points. This scoring algorithm is reversed for the unhealthy components. For alcohol, individuals with moderate intake (males: 10–50 g/day; females: 5–25 g/day) receive 1 point. Overall, the MedDiet score ranges from 0 (low adherence) to 9 (high adherence).

**Table 1:** Food items included in food groups contributing to the Mediterranean diet score.

Whole grains	Whole-grain bread, oat, barley, oatmeal
Fruits	Cantaloupe, honeydew melon, watermelon, apricot, cherries, peaches, prunes, strawberries, plums, figs, grapes, pears, apples, kiwifruit, citrus, pomegranate, banana, persimmon, date, dried fruits, raisin, fruit juices
Vegetables	Lettuce, cabbage, tomato, cucumber, leafy green, eggplant, celery, beet, carrot, garlic, onion, pepper, mushroom, green peas, green beans, zucchini, mixed vegetables
Nuts	Walnuts, peanut, other nuts, seeds
Legumes	Beans, chickpea, lentil, soybean, pea
Fish/Seafood	Fish, tuna
Meats	Red meat, chicken, processed meat

### 2.4. Anthropometric assessment

Weight was measured with participants wearing light clothing and without shoes using the InBody 770 (InBody Co, Seoul, Korea) machine. Height was measured without shoes in the standing position using the automatic stadiometer (BSM 370; Biospace Co, Seoul, Korea). Then, body mass index (BMI) was computed using Quetelet’s index from weight in kilograms divided by height in meters squared. BMI was categorized according to the World Health Organization criteria as underweight (<18.5 kg/m^2^), normal weight (18.5 to 24.9 kg/m^2^), overweight (25 to 29.9 kg/m^2^), and obesity (≥30 kg/m^2^). While the participants were in the standing position and at the end of gentle expiration, waist circumference (WC) was measured in the standing position at the minimum circumference between the iliac crest and the rib cage. Also, hip circumference (HC) was measured in the standing position at the maximum circumference of the buttocks.

### 2.5. Demographic data

Age (years), sex (male, female), marital status (married, single), education level (illiterate, elementary school, middle school, high school diploma, college degrees), regular consumption of dietary supplements (yes, no), smoking status (non-smoker, current smoker, former smoker, passive smoker), sleep duration (hours), physical activity level (low: 24–36.5 METs, medium: 36.6–44.9 METs, high: ≥45 METs), medication use (no, yes), diabetes mellitus (no, yes), hypertension (no, yes), cardiovascular diseases (no, yes) were collected at baseline by self-administered questionnaires. The single marital status referred to never married, widowed, and divorced. The dietary supplement’s item included multi-vitamin minerals, multi-vitamin, calcium, calcium + vitamin D, vitamin D, folic acid, omega 3 or fish oil, iron, and zinc.

### 2.6. Statistical analysis

All statistical analyses were conducted with the SPSS software. We also excluded subjects with missing data. The normal distribution of continuous data was ascertained using the Kolmogorov–Smirnov test. Data are presented as means ± standard deviations (SD) for normally distributed variables, medians [interquartile ranges] for non-normally distributed variables, and percentages (*n*%) for qualitative variables. We categorized the MedDiet score according to tertiles as high adherence (third tertile) and low adherence (first tertile). Differences across the tertiles of the MedDiet score were determined by the one-way analysis of variance (ANOVA) with the Bonferroni *post hoc* test and Kruskal-Wallis test for normally and non-normally distributed variables, respectively. The chi-square test was applied to determine the distribution of quantitative variables across the tertiles of MedDiet. Also, linear trends were assessed using the one-way ANOVA test and the Jonckheere-Terpstra test.

Binary logistic regression models (Enter Method) were used to estimate odds ratios (ORs) with 95% confidence intervals (95% CI) for having hepatic fibrosis across the tertiles of the MedDiet score. Three multivariate models were run to adjust possible confounding factors which were obtained from the comparison of demographic, anthropometric, and biochemical characteristics of participants across the tertiles of the Mediterranean diet scores (Tables 2 and 3) and our previous knowledge (21). We defined adjusted models as follows:

1. Model I was adjusted for sex (male, female), smoking status (non-smoker, current smoker, former smoker, passive smoker), physical activity levels (low, medium, high), educational status (illiterate, elementary school, middle school, high school diploma, college degrees), diabetes mellitus (yes, no), and using dietary supplements (yes, no).
2. Model II was adjusted for sex (male, female), smoking status (non-smoker, current smoker, former smoker, passive smoker), physical activity levels (low, medium, high), educational status (illiterate, elementary school, middle school, high school diploma, college degrees), diabetes mellitus (yes, no), and using dietary supplements (yes, no), weight, total cholesterol, and LDL-c.
3. Model III was adjusted for sex (male, female), smoking status (non-smoker, current smoker, former smoker, passive smoker), physical activity levels (low, medium, high), educational status (illiterate, elementary school, middle school, high school diploma, college degrees), diabetes mellitus (yes, no), and using dietary supplements (yes, no), weight, total cholesterol, and LDL-c, and FLI scores.

**Table 2:** Demographic characteristics of NAFLD (defined through the FLI) participants in the RaNCD cohort across the tertiles of the Mediterranean diet scores.

Variables		Tertiles (T) of Mediterranean diet score			*p*-value
		T_1_ *low adherence*	T_2_	T_3_ *high adherence*	
Mediterranean diet score		0–3	4–5	6–8	-
Number		1,462	1,284	579	-
Age; years		48.70 ± 8.04	48.02 ± 8.00	48.16 ± 8.13	0.169^*^
Male; *n*%		659 (45.1%)	534 (41.6%)	193 (33.3%)	<0.001
Married; *n*%		1,349 (92.3%)	1,193 (92.9%)	532 (91.9%)	0.695
Education	Illiterate; *n*%	811 (55.5%)	607 (47.3%)	273 (47.2%)	<0.001
	College degrees; *n*%	74 (5.1%)	103 (8%)	55 (9.5%)	
Hypertension; *n*%		299 (20.5%)	274 (21.3%)	123 (21.2%)	0.832
Diabetes mellitus; *n*%		195 (13.3%)	171 (13.3%)	104 (21.2%)	0.015
Cardiovascular diseases; *n*%		324 (22.2%)	323 (25.2%)	142 (24.5%)	0.163
Smoking	Current smoker; *n*%	134 (9.2%)	103 (8%)	21 (3.6%)	<0.001
	Former smoker; *n*%	115 (10.3%)	116 (9%)	42 (7.3%)	
	Non-smoker; *n*%	566 (38.7%)	548 (42.7%)	267 (46.1%)	
Physical activity	Low; *n*%	509 (34.8%)	477 (37.1%)	201 (34.7%)	0.001
	High; *n*%	263 (18%)	184 (14.3%)	64 (11.1%)	
Medication use; *n*%		432 (29.5%)	387 (30.1%)	184 (31.8%)	0.613
Dietary supplement use; *n*%		183 (12.5%)	101 (7.9%)	33 (5.7%)	<0.001
Sleep duration; hours		7.12 ± 1.23	7.00 ± 1.24	7.04 ± 1.15	0.122^*^

NAFLD, nonalcoholic fatty liver disease; FLI, fatty liver index. Values are shown as a mean ± standard deviation or frequencies.

* *p*-values were obtained using the chi-square test or one-way ANOVA test.

**Table 3:** Association of the Mediterranean diet scores with anthropometric and biochemical characteristics in patients with NAFLD defined through the FLI.

Variables	Tertiles of Mediterranean diet score			P-trend	Beta Coefficient (95% CI) per SD
	T_1_ *low adherence*	T_2_	T_3_ *high adherence*		
Weight; kg	82.25 ± 12.43	81.90 ± 11.59	80.02 ± 10.57	<0.001	−0.417 (−0.819 – −0.014)
BMI; kg.m^−2^	31.23 ± 4.37	31.17 ± 4.04	30.88 ± 3.65	0.078	−0.033 (−0.173–0.108)
WC; cm	105.89 ± 8.08	105.81 ± 8.32	105.35 ± 8.41	0.181	−0.124 (−0.404–0.156)
HC; cm	108.89 ± 8.20	109.18 ± 8.45	109.07 ± 8.72	0.661	0.192 (−0.039–0.478)
WHR	0.982 ± 0.053	0.982 ± 0.055	0.979 ± 0.053	0.430	0.001 (−0.001–0.002)
FBS; mg.dl^−1^	103.03 ± 32.77	102.51 ± 32.68	105.07 ± 35.58	0.211	0.693 (−0.438–1.823)
TC; mg.dl^−1^	199.09 ± 39.64	195.16 ± 39.28	193.69 ± 37.37	0.005	−2.505 (−3.835 – −1.175)
TG; mg.dl^−1^	159 [116–226]	157 [119–219]	155 [116–204]	0.210^*^	−2.852 (−6.223–0.518)
HDL; mg.dl^−1^	44.63 ± 10.84	43.42 ± 10.58	44.94 ± 10.58	0.546	−0.210 (−0.572–0.151)
LDL; mg.dl^−1^	110.56 ± 25.93	107.46 ± 25.46	106.57 ± 25.21	0.002	−1.932 (−2.803 – −1.061)
ALT; IU.L^−1^	24 [18–33]	24 [18–34]	23.9 [18–33]	0.999^*^	0.114 (−0.484–0.712)
AST; IU.L^−1^	20.6 [17–25]	20 [17–25]	19.5 [16–24]	0.176^*^	−0.244 (−0.598–0.109)
GGT; IU.L^−1^	25.6 [19–38]	25.4 [18–37]	24.8 [18–36]	0.111^*^	0.365 (−0.546–1.274)
ALP; IU.L^−1^	210.58 ± 62.51	205.98 ± 60.26	206.92 ± 60.00	0.224	−1.872 (−3.954–0.210)
Platelet; 10^3^/μl	253.37 ± 59.44	264.57 ± 64.05	269.56 ± 62.59	<0.001	7.489 (5.376–9.603)

ALP, alkaline phosphatase; ALT, alanine aminotransferase; AST, aspartate aminotransferase; BMI, body mass index; FBS, fasting blood sugar; FLI, Fatty liver index; GGT, gamma-glutamyltransferase; HC, hip circumference; TC, total cholesterol; TG, triglyceride; WC, waist circumference; WHR, waist to hip ratio. Values are shown mean ± standard deviation or mean (95% confidence interval). Beta coefficients (95 %CI) were obtained using the linear regression test.

* *p*-trends were obtained using the one-way ANOVA test and Jonckheere-Terpstra test.

We also applied univariate and multivariate linear regression models to determine changes in variables for each SD increase in the MedDiet score. *p*-values less than 0.05 were considered to indicate statistical significance.

## 3. Results

**Figure 1:**
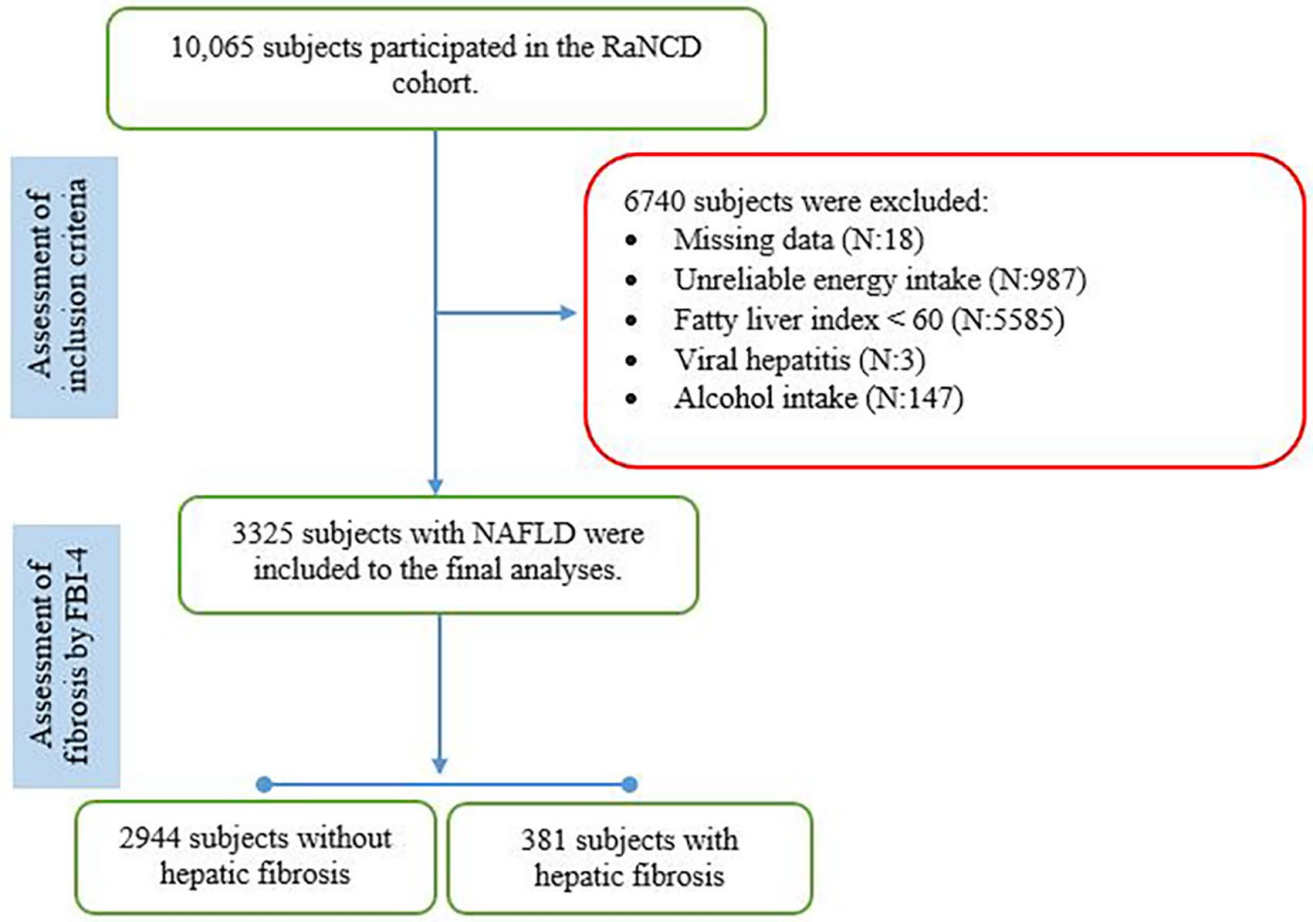
The flow chart of the study.

Subjects in the present study were recruited from the RaNCD cohort including 10,065 Kurdish participants, of whom 6,740 were excluded because of missing relevant data (n:18), implausible energy intake (n:987), FLI less than 60 (n:5587), alcohol intake (n:147), and hepatitis B (n:2). The final number of subjects included in the analyses was 3,325 (Figure 1). The mean (±SD) age was 48.34 (±8.05) years, BMI was 31.23 (±3.86) kg/m^2^, and FLI was 77.25 (±10.56), and 1939 subjects (58.3%) were female and 381 (11.5%) had hepatic fibrosis according to the FIB-4 index. Subjects with hepatic fibrosis were more likely to be male (15.8 vs. 8.4%; *p*-value: 0.001), intake the dietary supplement (16.1 vs. 11%; *p*-value: 0.007), and have lower BMI (30.43 ± 3.81 vs. 31.24 ± 4.16; *p*-value: 0.001) than those without hepatic fibrosis. Groups did not differ in terms of marital status, educational levels, smoking, medication use, diabetes mellitus, hypertension, cardiovascular diseases, sleep duration, physical activity levels, weight, WC, HC, and WHR (*p*-value > 0.05).

Table 2 shows the demographic characteristics of subjects by the tertiles of the MedDiet score. Subjects with higher adherence to the MedDiet (highest tertile) were more likely to be female and non-smokers, intake dietary supplements, and have diabetes mellitus and higher educational levels but lower physical activity than those with the lower adherence to the MedDiet (lowest tertile). Further analysis showed that the MedDiet score was significantly higher in females than males (mean difference: 0.28; 95% CI: 0.40–0.16), in subjects with a college’s degree than in illiterate (mean difference: 0.65; 95% CI: 0.31–0.98), in non-consumers of dietary supplements than consumers (mean difference: 0.75; 95% CI: 0.95–0.55), in subjects with low physical activity than high physical activity (mean difference: 0.35; 95% CI: 0.13–0.57), and in subjects with diabetes mellitus than others (mean difference: 0.16; 95% CI: 0.01–0.33). There were no significant differences between the MedDiet score and other demographic data (*p*-value > 0.05).

A significant linear trend was observed in FLI values across the tertiles of the MedDiet score (P-trend: <0.001). Subjects in the highest tertile of the MedDiet score had a lower FLI value than those in the lowest tertile (75.42 ± 10.11 vs. 77.61 ± 10.70; *p*-value: <0.001). The mean (±SD) of anthropometric and biochemical characteristics of participants across the tertiles of the MedDiet score are shown in Table 3. A significant linear trend was observed in weight, platelet count, total cholesterol (TC), and LDL-c across the tertiles of the MedDiet score. Subjects in the highest tertile of the MedDiet score had a higher platelet count and a lower weight, TC, and LDL-c than those in the lowest tertile (*p*-value < 0.05). The linear regression model showed that adherence to the MedDiet was associated with a 7.48 (95%CI: 5.376–9.603; *p*-value: 0.001) × 10^3^/μl increase in platelet count and 0.417 (95%CI: 0.819–0.014; *p*-value: 0.042) kg, 2.505 (95%CI: 3.835–1.175; *p*-value: 0.001) mg/dl and 1.93 (95%CI: 2.803–1.061; *p*-value: 0.001) mg/dl reduction in weight, TC, and LDL-c for each SD increase in the score, respectively.

Odds ratios (95% CIs) for having hepatic fibrosis by tertiles of the MedDiet score are shown in Table 4. A significant linear trend was observed in the odds of hepatic fibrosis across the tertiles of the MedDiet score. Subjects in the highest tertiles of the MedDiet score had lower odds of hepatic fibrosis than the lowest tertile (*p*-value: 0.004). This linear trend was attenuated but remained significant after the adjustment of the potential confounding factors related to demographic (model I), biochemical, anthropometric characteristics (model II), and severity of hepatic steatosis (model III). Further analysis showed that adherence to the MedDiet was associated with about 16% lower odds of having hepatic fibrosis in patients with NAFLD for each SD increase in the score. Regardless of the cut-off points of FIB-4 for having hepatic fibrosis, a significant relationship was observed between the FIB-4 values and the Mediterranean diet scores (Beta Coefficient: -0.023; 95%CI: −0.033 to-0.012).

**Table 4:** Association between the Mediterranean diet scores and hepatic fibrosis (defined through the FIB-4 score) in patients with NAFLD.

Models	Odds ratio (95% CI) across tertiles (T) of the Mediterranean diet score			P-trend	Odds ratio (95% CI) per SD
	T_1_ *low adherence*	T_2_	T_3_ *high adherence*		
Crude model	1.00 (ref.)	0.917 (0.729–1.154)	0.613 (0.438–0.857)	0.008	0.817 (0.734–0.910)
Adjusted model I	1.00 (ref.)	0.953 (0.755–1.202)	0.666 (0.474–0.937)	0.038	0.843 (0.755–0.942)
Adjusted model II	1.00 (ref.)	0.935 (0.740–1.181)	0.656 (0.466–0.923)	0.028	0.835 (0.747–0.933)
Adjusted model III	1.00 (ref.)	0.929 (0.735–1.173)	0.665 (0.472–0.937)	0.032	0.841 (0.752–0.940)

FLI, fatty liver index; FIB-4, fibrosis-4 score; SD, standard deviation. Values are shown mean (95% confidence interval). Model I: Adjusted for sex, smoking status, physical activity levels, educational status, diabetes mellitus, and using dietary supplements. Model II: Further adjusted for the weight, total cholesterol, and LDL cholesterol. Model III: Further adjusted for FLI scores. Odds ratios were obtained using the binary logistic regression test.

## 4. Discussion

Using data on 3,325 adult participants from the RaNCD cohort, we found that greater adherence to the MedDiet was associated with a lower risk of hepatic fibrosis, as measured by the FIB-4 score. In addition, our results provided supportive evidence for the benefits of adherence to the MedDiet in controlling weight, hepatic steatosis, and dyslipidemia. To our knowledge, this is the first large-scale study investigating the relationship between the MedDiet and hepatic fibrosis in the Iranian population.

The MedDiet emphasizes a high intake of whole grains, fruits, vegetables, legumes, nuts, and fish and a low intake of red and processed meats and dairy products. Our results showed that adherence to the MedDiet significantly reduces the risk of hepatic fibrosis in individuals with NAFLD. Despite differences in participating population, MedDiet capture, and methods of hepatic fibrosis measure; the findings of previous research are consistent with our results. Baseline data of the ATTICA cohort including 3,042 Greek adults showed that individuals in the highest tertile of the MedDiet score had a lower age, weight, FLI, FIB-4, and proportion of diabetes mellitus but higher physical activity than those in the lowest tertile (12). In a cross-sectional study conducted on 160 diabetic patients with NAFLD proven through biopsy, subjects with hepatic fibrosis had lower adherence to the MedDiet compared to those without fibrosis (13). A quasi-interventional study on 44 Greek Caucasian patients with non-fibrotic NAFLD showed that increased adherence to the MedDiet significantly associated with improvements in liver imaging, NAFLD fibrosis score (NFS), and C-reactive protein (22). Furthermore, exploratory dietary patterns almost similar to the MedDiet were associated with a lower risk of hepatic fibrosis. In this context, Soleimani et al. reported that adherence to a healthy dietary pattern that emphasizes high intakes of low-fat dairy products, vegetables, fruits, nuts, white meats (poultry and fish), and vegetable oils and low intakes of red and processed meats significantly related to lower odds of hepatic fibrosis in patients with NAFLD, whereas the adherence to a western dietary characterized by high intakes of red and processed meats, refined grains, potato, white meat, eggs, and soft drinks and low intakes of red and processed vegetables, fruits, and nuts related to the increased odds of hepatic fibrosis (21).

As expected, individuals with a higher adherence to the Mediterranean showed a better overall health profile including lower weight, TC, LDL-c, higher educational status, and better smoking status. This phenomenon could influence the relationship of the MedDiet with hepatic fibrosis. Our results when adjusted for confounding factors such as weight, liver fat count, TC, LDL-c, educational status, and smoking were attenuated but remained significant (multivariate adjusted OR per SD increment: 0.841; 95% CI: 0.752–0.940). Therefore, our results seemed to be more robust than the previous studies in controlling potential confounding factors (12, 13). Furthermore, more tendency toward healthier diets following intermediary events related to NAFLD such as dyslipidemia and diabetes would tend to weaken the relationship between the MedDiet and hepatic fibrosis (21).

Several potential mechanisms may explain the inverse association between adherence to the MedDiet and the risk of hepatic fibrosis in patients with NAFLD. Weight is considered an independent predictor for the progression of hepatic steatosis to fibrosis in NAFLD (23). It has been proven that weight loss leads to improve hepatic fibrosis in obese patients with NASH (24, 25). Our results are consistent with most studies that adherence to the MedDiet was significantly associated with better metabolic control, especially weight (26, 27). The high-fiber content in the MedDiet can protect individuals against overweight and obesity development by suppressing the person’s appetite sensation and reducing the diet’s calorie density along with glycemic index (28, 29). We observed that the association of the MedDiet with hepatic fibrosis remained significant after the adjustment of weight and other confounding factors. This observation implies that this diet might have a direct impact on liver fibrogenesis. Hepatic stellate cells (HSCs) are the main cells in hepatic fibrogenesis by synthesizing extracellular matrix proteins (ECMs), including collagen and fibronectins. In response to fibrogenic stimuli such as reactive oxygen species (ROS) and inflammatory cytokines, quiescent HSCs become active, secrete ECMs, and decrease their degradation (30). Activated HSCs also migrate to the site of damage and transdifferentiate into proliferative, contractile, and ECMs-secreting myofibroblasts (31). Nowadays, HSCs inhibitors have been considered a promising approach for hepatic fibrosis treatment (30, 31). The MedDiet contains large amounts of antioxidants such as polyphenols, carotenoids, vitamin C, vitamin E, and selenium that may act synergistically to reduce the production of pro-inflammatory mediators as well as ROS by inhibiting nuclear factor-κB (32, 33). Our recent trial showed that propolis)a honeybee product with high but diverse contents of polyphenols (significantly reduces inflammation and hepatic fibrosis in patients with NAFLD (34). In addition, the MedDiet emphasizes a low intake of SFAs–rich sources including red meats, processed meats, and dairy products. A high intake of SFAs is associated with the up-regulation of inflammatory-related genes (35). Altogether, the MedDiet may directly protect the liver from fibrosis which in part is due to its anti-inflammatory and antioxidative properties.

The major strengths of our study include the large sample size, population-based design, homogeneous participants, and use of different confounding factors in statistical models. Also, liner regression test regenerated the results of between-group analysis that imply the absence of miss classification across the tertiles of MDiet score in our study. However, the current study is not able to draw the causal relationship between the MedDiet and hepatic fibrosis because of the nature of the cross-sectional study design. It should also be noted that recall bias is a common error in retrospective studies (like our study), especially when the control group and the case group do not have the same behavior in remembering the exposure factors such as diet. Additionally, undesirable habits such as eating unhealthy foods tend to be under-reported, and are therefore subject to recall bias. In the current study to decline the recall bias, the FFQ was taken from the participants by trained nutrition experts, and the participants were given enough time to remember the consumption of each food item. Therefore, the longitudinal study design and clinical trial are warranted to overcome the recall bias and elucidate the causal relationship of the MedDiet and its components with the risk of hepatic fibrosis in NAFLD. Another limitation of this study is the likely presence of measurement error due to the use of FFQ. This questionnaire may not accurately measure the exact intake of foods and nutrients but it is a validated instrument for investigating diet-disease relations in epidemiological studies (36). Furthermore, our findings are not generalizable to all adults because the RaNCD study only includes patients 35–65 years old (as a limitation) and to individuals with other causes of hepatic steatosis than NAFLD because of the different nature of their pathogenesis in hepatic fibrosis.

## 5. Conclusion

The current investigation that adherence to the MedDiet characterized by a high intake of whole grains, fruits, vegetables, legumes, nuts, and fish was significantly associated with a lower risk of having hepatic fibrosis in patients with NAFLD. Further studies are required to elucidate mechanisms and the causal relationship of observed association in individuals of all ages, ethnicities, and etiologies of hepatic steatosis.

## Data availability statement

The data analyzed in this study is subject to the following licenses/restrictions: The data presented in this study are available on request from the steering committee of the RaNCD. The data are not publicly available as this is an ongoing cohort study. Requests to access these datasets should be directed to YP; Email: yahya.pasdar@kums.ac.ir.

## Ethics statement

The studies involving human participants were reviewed and approved by the ethics committee of Kermanshah University of Medical Sciences (IR.KUMS.REC.1401.223). The patients/participants provided their written informed consent to participate in this study.

## Author contributions

MMi, MD, DS, and MMo wrote the main manuscript text. FN and YP administrated project. All authors contributed to the article and approved the submitted version.

## Funding

We sincerely appreciate all participants and the Student Research Committee, Kermanshah University of Medical Sciences, Kermanshah, Iran (IR.KUMS.REC.1401.223) and PERSIAN cohort Study collaborators for their financial support of the study. The Iranian Ministry of Health and Medical Education has contributed to the funding used in the PERSIAN Cohort through Grant no 700/534.

## Conflict of interest

The authors declare that the research was conducted in the absence of any commercial or financial relationships that could be construed as a potential conflict of interest.

## Publisher’s note

All claims expressed in this article are solely those of the authors and do not necessarily represent those of their affiliated organizations, or those of the publisher, the editors and the reviewers. Any product that may be evaluated in this article, or claim that may be made by its manufacturer, is not guaranteed or endorsed by the publisher.
